# Supplementary material for: From sequence to enzyme mechanism using multi-label machine learning
Source: BMC Bioinformatics. 2014 May 19;15:150. doi: 10.1186/1471-2105-15-150 (PMC4229970; doi:10.1186/1471-2105-15-150)
Supplement: Additional file 2 — Java code of ml2db. Additional file ml2db_code.tar.gz contains the Java source code to run the multi-label machine learning experiments and save the results to database. The code’s Javadoc is included. [file 1471-2105-15-150-S2.zip › additional file 2/ml2db/ml2db/doc/main/ml2db/al/utility/UtilityMetric.html]

UtilityMetric


JavaScript is disabled on your browser.


- Overview
- Package
- Class
- Use
- Tree
- Deprecated
- Index
- Help

- Prev Class
- Next Class

- Frames
- No Frames

- All Classes

- Summary:
- Nested |
- Field |
- Constr |
- Method

- Detail:
- Field |
- Constr |
- Method


main.ml2db.al.utility

## Class UtilityMetric

- java.lang.Object
- - main.ml2db.al.utility.UtilityMetric

- Direct Known Subclasses:
  :   ConfidenceMetric, GlobalDistanceMetric, NeighbourhoodNetwork, RandomPicker

  ---

    

  ```
  public abstract class UtilityMetric
  extends java.lang.Object
  ```

  A generic utility metric to pick the next (query) instance for active
  learning

  Version:
  :   20 Jul 2011

  Author:
  :   Luna De Ferrari ldf(at)st-andrews.ac.uk

- - ### Constructor Summary

    Constructors

    Constructor and Description

    `UtilityMetric()`
  - ### Method Summary

    Methods

    | Modifier and Type | Method and Description |
    | --- | --- |
    | `abstract java.lang.String` | `getMetricName()` |
    | `IdMultiLabelInstances` | `getUnlabelled()` |
    | `abstract double` | `getUtility(mulan.classifier.MultiLabelLearner trainedLearner, IdInstance instance)` |
    | `abstract void` | `initialise()` |
    | `abstract void` | `refresh(IdMultiLabelInstances labelled, IdMultiLabelInstances unlabelled, IdInstance queryInstance)` Refresh the set of labelled and unlabelled instances |
    | `void` | `setUnlabelled(IdMultiLabelInstances unlabelledInstances)` |

    - ### Methods inherited from class java.lang.Object

      `equals, getClass, hashCode, notify, notifyAll, toString, wait, wait, wait`

- - ### Constructor Detail


    - #### UtilityMetric

      ```
      public UtilityMetric()
      ```
  - ### Method Detail


    - #### getMetricName

      ```
      public abstract java.lang.String getMetricName()
      ```


    - #### getUnlabelled

      ```
      public IdMultiLabelInstances getUnlabelled()
      ```


    - #### getUtility

      ```
      public abstract double getUtility(mulan.classifier.MultiLabelLearner trainedLearner,
                      IdInstance instance)
      ```


    - #### initialise

      ```
      public abstract void initialise()
      ```


    - #### refresh

      ```
      public abstract void refresh(IdMultiLabelInstances labelled,
                 IdMultiLabelInstances unlabelled,
                 IdInstance queryInstance)
      ```

      Refresh the set of labelled and unlabelled instances

      Parameters:
      :   `labelled` -
      :   `unlabelled` -
      :   `queryInstance` -


    - #### setUnlabelled

      ```
      public void setUnlabelled(IdMultiLabelInstances unlabelledInstances)
      ```


- Overview
- Package
- Class
- Use
- Tree
- Deprecated
- Index
- Help

- Prev Class
- Next Class

- Frames
- No Frames

- All Classes

- Summary:
- Nested |
- Field |
- Constr |
- Method

- Detail:
- Field |
- Constr |
- Method
